# Association of race and ethnicity with postpartum contraceptive method choice, receipt, and subsequent pregnancy

**DOI:** 10.1186/s12905-020-01162-8

**Published:** 2021-01-07

**Authors:** David Ngendahimana, Jessica Amalraj, Barbara Wilkinson, Emily Verbus, Mary Montague, Jane Morris, Kavita Shah Arora

**Affiliations:** 1 Case Western Reserve University School of Medicine, Mary Ann Swetland Center for Environmental Health, Cleveland, OH USA; 2 Department of Bioethics, Case Western Reserve University School of Medicine, Cleveland, OH USA; 3 Case Western Reserve University School of Medicine, Cleveland, OH USA; 4 Department of Obstetrics and Gynecology, MetroHealth Medical Center, Case Western Reserve University, 2500 MetroHealth Drive, Cleveland, OH 44109 USA

**Keywords:** Postpartum contraception, Disparity, Race/ethnicity, Long-acting reversible contraception, Implicit bias, Contraceptive counseling

## Abstract

**Background:**

We sought to assess racial/ethnic differences in choice of postpartum contraceptive method after accounting for clinical and demographic correlates of contraceptive use.

**Methods:**

This is a secondary analysis of a single-center retrospective cohort study examining postpartum women from 2012 to 2014. We determined the association between self-identified race/ethnicity and desired postpartum contraception, receipt, time to receipt, postpartum visit attendance, and subsequent pregnancy within 365 days of delivery.

**Results:**

Of the 8649 deliveries in this study, 46% were by Black women, 36% White women, 12% Hispanic, and 6% by women of other races. Compared with White women, Black and Hispanic women were more likely to have a postpartum contraception plan for all methods. After multivariable analysis, Hispanic women (relative to White women) were less likely to receive their chosen method (odds ratio [OR] 0.74, 95% confidence interval [CI] 0.64–0.87). Women of races other than Black or Hispanic were less likely to experience a delay in receipt of their desired highly-effective method compared to White women (hazard ratio [HR] = 0.70, 95% CI 0.52–0.94). There were no differences between racial/ethnic groups in terms of postpartum visit adherence. Black women were more likely to be diagnosed with a subsequent pregnancy compared to White women (OR 1.17, 95% CI 1.04–1.32).

**Conclusion:**

Racial/ethnic variation in postpartum contraceptive outcomes persists after accounting for clinical and demographic differences. While intrinsic patient-level differences in contraceptive preferences should be better understood and respected, clinicians should take steps to ensure that the observed differences in postpartum contraceptive plan methods between racial/ethnic groups are not due to biased counseling.

## Background

Racial and ethnic disparities are pervasive in women’s healthcare and health outcomes in the United States [1]. Disparities in contraceptive use are especially troubling given the United States’ history of valuing reproduction based on a woman’s race and/or class, the importance of family planning in attaining personal educational and workforce goals, and the downstream impact on maternal and child health by reduction of short interpregnancy intervals [2–4]. However, Black and Hispanic women are reported to have higher rates of contraceptive non-use, differences in contraceptive method choice, lower rates of method fulfillment, contraceptive failure, and unintended pregnancies than White women [5–8].

However, the cause of this disparity is unclear and likely multifactorial including differences at the patient-level, clinician-level such as implicit bias, and societal level including structural racism and barriers to care [1]. At the patient-level, variation in patient preferences including individual intention to conceive or seek pregnancy may contribute to differences in contraceptive method chosen [9, 10]. Demographic and clinical factors that are correlates of contraceptive use such as age, parity, and insurance status also vary by race/ethnicity [11]. For example, Black and Hispanic women are more likely to utilize less effective methods of contraception than White women, but also more likely to utilize the highly effective method of sterilization—a relationship which is also associated with age [12, 13]. Prior assessments of differences in contraceptive method choice by race/ethnicity largely did not take into account the potential impact of all of these clinical and demographic factors [5, 6, 8–10, 14, 15].

Therefore, the primary objective of this study was to assess self-identified racial/ethnic differences in choice of postpartum contraceptive method after accounting for clinical and demographic correlates of contraceptive use. We also sought to analyze racial/ethnic variation in postpartum contraceptive method fulfillment, time to fulfillment, postpartum visit attendance, and subsequent pregnancy. Given differences in contraceptive method used outside of the postpartum period, we hypothesized that Black and Hispanic women would be more likely to plan on using less-effective methods or sterilization after delivery, while White women would be more likely to plan on using a long-acting reversible contraception (LARC) method [6, 8]. Furthermore, given structural barriers to care and known disparities in access, we also hypothesized Black and Hispanic women would be less likely to have their contraceptive method choice fulfilled, more likely to experience delays in receipt, less likely to attend postpartum care, and more likely to be diagnosed with a subsequent pregnancy within one year of delivery [1].

## Methods

This is a planned secondary analysis of a retrospective cohort study of all deliveries at or above 20 weeks gestation at our academic, urban, tertiary care teaching hospital from January 1, 2012 through December 31, 2014. While the primary analysis restricted analysis of differences in postpartum sterilization fulfillment solely between women with Medicaid versus private insurance, the cohort for this secondary analysis was broadened to include all patients in the study cohort. Exclusion criteria was maternal death during the delivery hospitalization and prior sterilization with use of in vitro fertilization for the index pregnancy as a contraceptive method has already been fulfilled. Full study methodology has been published previously [16]. Briefly, we reviewed each patient’s linked outpatient and inpatient electronic medical record for demographic and clinical characteristics as well as study outcomes. Insurance status was directly obtained by matching patient charts to billing records.

Race/ethnicity was self-identified as the single best choice by the patient and analyzed as either White, Black, Hispanic, or Other. The primary study outcome was choice of postpartum contraceptive method (defined as the plan documented in the hospital discharge summary or, if not included in the discharge summary, in the last inpatient postpartum progress note). Contraceptive plan is a required field in our discharge summary. In rare cases, the clinician completing the discharge summary erased this field from the note and in those instances, the last inpatient postpartum progress note was utilized to abstract the documented contraceptive plan. All study subjects had a contraceptive plan documented in one of these two places. For the 732 (8.5% of the study cohort) of subjects who had two or more planned contraceptive methods, the most effective contraceptive method was selected for analysis.

Secondary outcomes included receipt of the desired contraceptive method, time to receipt of desired contraceptive method within 90 days of delivery, attendance at an outpatient postpartum visit within 90 days of delivery, and subsequent pregnancy within 365 days of delivery. Desired contraceptive method was consolidated into six groups—sterilization, LARC, combined hormonal contraception, Depo-Provera, norethindrone, and none. “None” was the reference category and included abstinence as well as those who specifically declined postpartum contraception. We calculated tests of differences in demographic and clinical characteristics across racial/ethnic groups with one-way analysis of variance (ANOVA) tests for continuous variables, χ^2^ with Yates’ continuity correction for categorical variables, and Fisher exact test for situations where counts were less than 5. We estimated odds ratios (OR) with 95% confidence intervals (CI) for postpartum contraceptive method planned and method receipt across races via unadjusted and adjusted multinomial logistic regression. Covariates included age, parity at admission, gestational age, prenatal visits, route of delivery, marital status, education level, and insurance. Variance Inflation Factors (VIF) were estimated for each covariates and the variable adjusted for if the VIF was below 10.

Differences in time to method receipt with 95% CIs across race/ethnicity were calculated using multivariable Cox proportional hazards regression stratified by highly-effective (sterilization or LARC) or moderately-effective (combined hormonal contraception, Depo-Provera, norethindrone) method for those women who received their contraceptive plans. Postpartum visit attendance and subsequent pregnancy were compared across the four race/ethnicity groups using the χ^2^ test. Subsequent pregnancy was defined as either positive urine or serum pregnancy test, presentation for prenatal care, or notation of pregnancy care at an outside hospital in our hospital’s clinical documentation.

Records were abstracted by four trained researchers (JM, BW, EV, MM) who coded in an iterative process to clarify interpretation of chart documentation. Complete data were available for 7613 (88%) records. All data available was analyzed. All analyses were performed using R version 3.5.3 using two-tailed tests at 0.05 significance level. Inpatient postpartum LARC was not available at our hospital (or any in the region) at the time of the study. Inpatient sterilization after vaginal delivery was available at our hospital. In our state, public and private insurance covers the cost of all contraceptive methods with zero cost-sharing on the part of the patient. This study was approved by the institutional review board of MetroHealth Medical Center.

## Results

After accounting for five exclusions due to the criteria above, 8649 deliveries remained. Of these, 46% were by Black women, 36% White women, 12% Hispanic, and 6% by women of other races. Clinical and demographic characteristics across race/ethnicity are presented in Table 1. Briefly, Black women tended to be younger, have higher parity, deliver at an earlier gestational age, have fewer prenatal visits, be unmarried, and be publicly insured. White women were more likely to be nulliparous, have attended college, and have private insurance. Hispanic women had more prenatal visits and were less likely to have attended college.

**Table 1:** Clinical and demographic characteristics

	Black (n = 4013)	White (n = 3072)	Hispanic (n = 1026)	Other (n = 538)	*p* test
Age (years)	28.81 (5.95)	30.72 (6.00)	29.27 (6.03)	31.20 (5.97)	< 0.001
Parity at admission					< 0.001
0	1385 (34.5)	1292 (42.1)	342 (33.3)	220 (40.9)	
1	1044 (26.0)	897 (29.2)	302 (29.4)	184 (34.2)	
2+	1584 (39.5)	883 (28.7)	382 (37.2)	134 (24.9)	
Gestational age at delivery (weeks)	38.04 (3.06)	38.29 (2.76)	38.33 (2.65)	38.45 (2.59)	< 0.001
Prenatal visits	8.52 (4.03)	9.50 (3.92)	9.82 (3.83)	8.97 (3.66)	< 0.001
Delivery type					< 0.001
Cesarean section	1035 (25.8)	787 (25.6)	265 (25.8)	117 (21.7)	
Operative vaginal delivery	108 (2.7)	112 (3.6)	34 (3.3)	35 (6.5)	
Spontaneous vaginal delivery	2870 (71.5)	2173 (70.7)	727 (70.9)	386 (71.7)	
Married					< 0.001
Yes	313 (7.8)	1069 (34.8)	219 (21.3)	301 (55.9)	
No	3598 (89.7)	1939 (63.1)	783 (76.3)	231 (42.9)	
Missing	102 (2.5)	64 (2.1)	24 (2.3)	6 (1.1)	
Education level					< 0.001
Attended college	1002 (25.0)	1289 (42.0)	212 (20.7)	221 (41.1)	
No college	2876 (71.7)	1663 (54.1)	764 (74.5)	287 (53.3)	
Missing	135 (3.4)	120 (3.9)	50 (4.9)	30 (5.6)	
Insurance					< 0.001
Public	3584 (89.3)	2108 (68.6)	875 (85.2)	355 (66.0)	
Private	316 (7.9)	848 (27.6)	90 (8.8)	120 (22.3)	
None	112 (2.8)	116 (3.8)	61 (5.9)	63 (11.7)	
Missing	1 (0.0)	0 (0.0)	0 (0.0)	0 (0.0)	
Contraceptive plan					< 0.001
Abstinence	19 (0.5)	36 (1.2)	4 (0.4)	9 (1.7)	
Copper IUD	17 (0.4)	10 (0.3)	2 (0.2)	3 (0.6)	
Declined	546 (13.4)	904 (29.4)	168 (16.4)	194 (36.0)	
Depo-Provera	1387 (34.6)	562 (18.3)	243 (23.7)	83 (15.4)	
IUD (general)	410 (10.2)	256 (8.3)	129 (12.6)	51 ( 9.5)	
Levonorgestrel IUD	47 (1.2)	33 (1.1)	18 (1.8)	7 (1.3)	
Norethindrone	578 (14.4)	460 (15.0)	146 (14.2)	60 (11.2)	
Combined Oral Contraceptive Pill	207 (5.2)	182 (5.9)	49 (4.8)	27 (5.0)	
Other	5 (0.1)	8 (0.3)	0 (0.0)	3 (0.6)	
Patch	8 (0.2)	5 (0.2)	2 (0.2)	0 (0.0)	
Subcutaneous implant	34 (0.8)	25 (0.8)	12 (1.2)	1 (0.2)	
Sterilization	667 (16.6)	402 (13.1)	213 (20.8)	51 (9.5)	
Vaginal ring	24 (0.6)	24 (0.8)	7 (0.7)	8 (1.5)	
Missing	70 (1.7)	165 (5.4)	33 (3.2)	41 (7.6)	

Presented as n (%) or mean (SD)

*IUD* intrauterine device

Black and Hispanic women planned to use Depo-Provera more often than any other method of contraception, whereas White women were more likely to decline contraception than use another method. Sterilization was most often selected as the postpartum contraceptive plan by Hispanic women (Table 1). Univariable and multivariable associations between race and planned postpartum contraceptive method are shown in Table 2. Compared with White women, Black and Hispanic women were more likely to have a documented postpartum contraception plan at time of hospital discharge for all contraception methods, though not statistically significant for combined hormonal contraception. Women of Other race were less likely to choose to use norethindrone (OR 0.59, 95% CI 0.42–0.83) or sterilization (OR 0.58, 95% CI 0.39–0.86), after adjusting for potential confounders.

**Table 2:** Univariable and multivariable associations of race/ethnicity and documented postpartum contraception plan^a^

Contraceptive method received	Black		Hispanic		Other	
	Unadjusted OR [95% CI]	Adjusted OR [95% CI]	Unadjusted OR [95% CI]	Adjusted OR [95% CI]	Unadjusted OR [95% CI]	Adjusted OR [95% CI]
Depo-Provera	3.74 [3.20, 4.38]	2.29 [1.91, 2.73]	2.17 [1.70, 2.76]	1.67 [1.28, 2.18]	0.58 [0.44, 0.77]	0.75 [0.54, 1.04]
LARC	2.40 [1.99, 2.90]	1.67 [1.35, 2.06]	2.50 [1.90, 3.28]	1.94 [1.43, 2.63]	0.80 [0.58, 1.11]	1.05 [0.74, 1.50]
Norethindrone	1.91 [1.60, 2.26]	1.43 [1.18, 1.73]	1.59 [1.22, 2.07]	1.38 [1.04, 1.84]	0.51 [0.37, 0.70]	0.59 [0.42, 0.83]
Combined Hormonal Contraception^b^	1.72 [1.38, 2.14]	1.21 [0.95, 1.55]	1.38 [0.98, 1.95]	1.17 [0.81, 1.70]	0.65 [0.44, 0.97]	0.83 [0.54, 1.26]
Sterilization	2.52 [2.11, 2.99]	1.52 [1.23, 1.87]	2.66 [2.07, 3.42]	2.24 [1.67, 3.00]	0.50 [0.36, 0.70]	0.58 [0.39, 0.86]

*OR* odds ratio, *CI* confidence interval

^a^Referent race—White. Covariates included age, parity, gestational age, prenatal visits, route of delivery, marital status, education level, and insurance

^b^Combined oral contraceptive pill, patch, vaginal ring

Table 3 shows univariable and multivariable analyses of the associations between race and receipt of the desired contraceptive plan, postpartum visit adherence and subsequent pregnancy. After adjusting for potential confounders, Hispanic women (relative to White women) were less likely to receive their chosen contraceptive plan (OR 0.74, 95% CI 0.64–0.87). There were no differences between racial/ethnic groups in terms of postpartum visit adherence. Black women were more likely to be diagnosed with a subsequent pregnancy within 365 days of delivery compared to White women (OR 1.17, 95% CI 1.04–1.32).

**Table 3:** Univariable and multivariable associations of postpartum contraception plan receipt, postpartum visit adherence, and subsequent pregnancy and race/ethnicity^a^

	Unadjusted effects			Adjusted effects^a^		
	Contraceptive receipt OR (95% CI)	Postpartum visit adherence OR (95% CI)	Subsequent pregnancy OR (95% CI)	Contraceptive receipt OR (95% CI)	Postpartum visit adherence OR (95% CI)	Subsequent pregnancy OR (95% CI)
Black	0.88 (0.79, 0.98)	0.65 (0.58, 0.71)	1.28 (1.15, 1.43)	1.01 (0.90, 1.14)	1.01 (0.90, 1.14)	1.17 (1.04, 1.32)
Hispanic	0.74 (0.64, 0.87)	0.85 (0.73, 0.99)	1.31 (1.12, 1.53)	0.82 (0.69, 0.97)	1.01 (0.85, 1.20)	1.14 (0.96, 1.36)
Other	1.13 (0.91, 1.41)	1.14 (0.93, 1.41)	1.10 (0.89, 1.35)	1.02 (0.81, 1.29)	1.18 (0.93, 1.50)	1.1 (0.87, 1.37)

^a^Referent race—White. Covariates included age, parity, gestational age, prenatal visits, route of delivery, marital status, education level, and insurance

Time to receipt of the desired postpartum contraceptive plan is shown in Table 4. A total of 6378 women were considered with 2316 receiving a highly-effective method and 4062 receiving a moderately-effective method. Overall, 75% of White, 78% of Black, and 74% of Hispanic women received their desired contraceptive method within 90 days of delivery. For highly-effective contraceptive methods, median times to receipt were 72 days [95% CI, 65–90 days] for Whites, 79 days [95% CI, 66–90 days] for Black and 70 days [95% CI, 57–80 days] for Hispanics. After multivariable analysis, women of Other races (other than Black or Hispanic) compared to White women were less likely to experience a delay in receipt of their desired highly-effective contraceptive method (HR = 0.70, 95% CI 0.52–0.94). The median time to receipt of a moderately-effective contraceptive method for all races was 2 days and there were no significant differences between Whites and other racial groups in terms of time to receipt of moderately-effective contraceptive methods.

**Table 4:** Cox proportional hazards analysis of time to receipt of desired postpartum contraceptive method by race/ethnicity

	Highly-effective plans		Moderately-effective plans	
	Unadjusted HR [95% CI]	Adjusted HR [95% CI]	Unadjusted HR [95% CI]	Adjusted HR [95% CI]
Black^a^	0.98 [0.87, 1.10]	0.98 [0.85, 1.12]	1.16 [1.08, 1.25]	1.08 [1.00, 1.17]
Hispanic^a^	1.09 [0.93, 1.29]	1.08 [0.90, 1.29]	1.01 [0.89, 1.12]	0.95 [0.84, 1.07]
Other	0.76 [0.57, 1.07]	0.70 [0.52, 0.94]	0.91 [0.77, 1.08]	0.93 [0.78, 1.12]

^a^Referent group—White women. Covariates included age, parity, gestational age, prenatal visits, route of delivery, marital status, education level, and insurance

## Discussion

After accounting for clinical and demographic differences between racial/ethnic groups that may impact contraceptive decision-making and use, we found that racial/ethnic variation in choice of postpartum contraceptive method exists. However, contrary to our hypothesis, Black and Hispanic women were more—not less—likely to choose every method of contraception (though not statistically significant for combined hormonal contraception) than White women for use as postpartum contraception. Little is known about racial/ethnic differences in planned method of postpartum contraception as most prior studies focus on contraceptive use, rather than plan [5–8]. Our study demonstrates that Black and Hispanic women plan on using contraception postpartum more often than their White counterparts. This finding is in contrast to a prior smaller study demonstrating little racial/ethnic variation in postpartum contraceptive plan [15]. However, given our study’s methodology, we are unable to determine the cause of this observed difference between racial/ethnic groups. While intrinsic patient-level differences in contraceptive preferences should be better understood and respected, clinicians should take steps to ensure that the observed differences in postpartum contraceptive plan methods between racial/ethnic groups are not due to biased counseling. Given bias in contraceptive counseling by race/ethnicity has been previously reported, interventions such as standardized counseling may help isolate individual patient preferences that may be mediated by cultural practices from the impact of subtly coercive behavior on the part of the clinician [17, 18].

In terms of our hypothesis regarding racial disparities in contraceptive fulfillment, we found that Hispanic, but not Black, women experienced a disparity in fulfillment of the chosen postpartum contraceptive method. Our findings provide nuance to the previously reported findings that Black and Hispanic women are less likely to use contraception. For Black women, this association was not demonstrated once we accounted for relevant clinical and demographic factors that impact contraceptive use. There were also no racial/ethnic differences in time to contraceptive receipt, as we had hypothesized. While there Black/Hispanic women were less likely to attend postpartum care in our univariable models, this association was no longer significant after multivariable analysis. This suggests that markers of access to healthcare such as insurance status are more strongly associated with postpartum care attendance [16].

Our final hypothesis was regarding disparities in subsequent pregnancy. Black, but not Hispanic, women were more likely to become pregnant within one year of index delivery despite the lack of difference in postpartum contraceptive receipt. This may be due to differences in ongoing access to short-acting methods of contraception, greater rates of discontinuation, or variations in desire for subsequent pregnancy [19–21]. While we did not compare Black versus Hispanic women, Hispanic women tended to pick more effective methods such as sterilization and LARC methods more often than Black women, perhaps contributing to the differences in subsequent pregnancy rates. Furthermore, strategies to improve access to the chosen method of postpartum contraception such as inpatient placement of LARC and enhanced access to postpartum sterilization services may benefit all women, but especially Hispanic women given their greater reliance on these methods and lower likelihood of receipt in our study [22, 23].

The major strength of this study is that it is a planned secondary analysis from a detailed and large data set including all deliveries over a 3-year period. This study has several limitations. The study is retrospective and is therefore limited by data available in and the quality of the medical records. As a single institution study, our patient demographics, clinician practices, and institutional policies surrounding sterilization may impact generalizability. Finally, several associations in our multivariable models are within the zone of potential bias and therefore should be interpreted with caution [24].

## Conclusions

In conclusion, race and ethnicity are factors that are independently associated with postpartum contraceptive method plan. Improved understanding of patient preferences is necessary to facilitate shared decision-making and enhance provision. Future study on differences in clinical counseling based on race/ethnicity is imperative in order to ensure such variation is autonomous.

## Data Availability

The datasets used and or/analyzed during the current study are not publicly available due to patient privacy concerns but are available from the corresponding author on reasonable request after IRB permission is obtained.

## Abbreviations

ANOVA: Analysis of variance
CI: Confidence interval
HR: Hazard ratio
LARC: Long-acting reversible contraception
OR: Odds ratio
VIF: Variance inflation factors
